# Influence of Neighborhood Socioeconomic Deprivation on Effectiveness of an Intensive Lifestyle Intervention

**DOI:** 10.1007/s11606-024-09232-5

**Published:** 2025-01-02

**Authors:** Mamadou Sy, Scott Pilla, Wendy Bennett, Hsin-Chieh Yeh, Kesha Baptiste-Roberts, Tiffany L. Gary-Webb, Dhananjay Vaidya, Jeanne M. Clark

**Affiliations:** 1 Department of Global and Community Health, College of Public Health, George Mason University, Fairfax, VA USA; 2 Department of Medicine, Division of General Internal Medicine, Johns Hopkins School of Medicine, Baltimore, MD USA; 3 Welch Center for Prevention, Epidemiology and Clinical Research, Baltimore, MD USA; 4 Department of Population, Family and Reproductive Health, Johns Hopkins Bloomberg School of Public Health, Baltimore, MD USA; 5 Department of Epidemiology, Johns Hopkins Bloomberg School of Public Health, Baltimore, MD USA; 6 Department of Public & Allied Health, School of Community Health & Policy, Morgan State University, Baltimore, MD USA; 7 Department of Epidemiology, School of Public Health, University of Pittsburgh, Pittsburgh, PA USA; 8 Johns Hopkins Brancati Center for Advancement of Community Care, Baltimore, USA; 9 Johns Hopkins Center for Health Equity, Baltimore, USA

**Keywords:** type 2 diabetes, obesity, lifestyle intervention, neighborhood deprivation, older adults

## Abstract

**Objective:**

To assess the influence of neighborhood socioeconomic deprivation on the effectiveness of an intensive lifestyle intervention (ILI) in the Look AHEAD trial.

**Research Design and Methods:**

Look AHEAD randomized adults with overweight/obesity and type 2 diabetes to ILI for weight loss, or Diabetes Support and Education (DSE). We linked participant data from four study sites to the 2000 United States Census to generate a neighborhood socioeconomic deprivation score. We analyzed the effect of neighborhood deprivation in tertiles on various clinical outcomes including weight and HbA1c changes over 4 years using a mixed-effects linear model with random intercept and an interaction term between deprivation tertile and study arm over 4 years.

**Results:**

Among 1213 participants at baseline, the mean age was 60 years, 41% were male, and 65% identified as White, 26% as Black, and 4% as Hispanic. Most participants had a college degree (84%) and reported an annual income over $40,000 (75%). The deprivation score ranged from −12.04 to −2.61 in the most deprived tertile and 2.01 to 18.69 in the least deprived tertile (the lower the score, the higher the deprivation). There were no statistically significant treatment differences by deprivation score in weight or HbA1c changes over the 4-year period.

**Conclusions:**

In this clinical trial population, an intensive lifestyle intervention was equally effective across levels of neighborhood socioeconomic deprivation. However, these findings may not extend to individuals with the lowest income and educational attainment who are not typically represented in clinical trials and for whom more research is needed.

**Supplementary Information:**

The online version contains supplementary material available at 10.1007/s11606-024-09232-5.

## INTRODUCTION

Diabetes prevalence in the USA continues to increase,^1,2^ with nearly 9% of the population currently diagnosed.^3^ Notably, 90% of these cases are attributed to type 2 diabetes.^4^ The risk of developing type 2 diabetes and its complications is heightened in individuals with overweight or obesity.^5,6^ Despite advances in diabetes treatment, the prevalence of poor glycemic control remains common,^1,2^ with only 50.5% of adults with diabetes achieving HbA1c levels below 7.0%.^1^

Individuals with diabetes living in areas with high deprivation are less likely to achieve glycemic control and more prone to develop complications.^7^ Deprived neighborhoods often lack access to healthy food^8^ and have fewer opportunities for exercise,^9^ which contribute to poor glycemic control.^10^ These factors may interact with individual-level socioeconomic factors such as education, income, and employment as underlying causes of poor diabetes outcomes.^7,11^

Lifestyle interventions, including behavior modification and physical activity, are recommended to improve glycemia and cardiometabolic risk.^12–15^ However, whether neighborhood deprivation moderates the effect of a lifestyle intervention on clinical measures such as blood sugar, body weight, lipids, or blood pressure is unknown. While some studies have examined this interaction, they mainly focused mainly on physical activity^16,17^ and weight loss,^18,19^ without reporting the impact on glycemic control.

Therefore, we conducted a secondary analysis of the Look AHEAD trial to explore whether neighborhood deprivation influences the effects of an intensive lifestyle intervention (ILI) on clinical outcomes (HbA1c, weight, blood pressure, lipid) in adults with overweight and type 2 diabetes. The Look AHEAD study was a multi-center randomized controlled trial examining the long-term effects of ILI on cardiovascular morbidity and mortality in individuals with overweight/obesity and diabetes.^20^ We hypothesized that participants living in more deprived neighborhoods would experience less improvement in clinical outcomes from the ILI.

## METHODS

This manuscript is based on an ancillary study of the Look AHEAD trial.^21,22^ The Look AHEAD trial has been described and reported on previously.^23^ Briefly, it was a randomized trial which tested the long-term effects of an ILI focused on weight loss in 5145 middle-aged and older adults with overweight or obesity and type 2 diabetes, who were recruited and randomized between 2001 and 2004. The ILI was delivered for a median of 9.6 years in 16 sites in the United States until it was terminated for futility for the primary outcome.^24^ The ILI aimed to induce a mean weight loss ≥ 7% of initial weight. Participants in the ILI group attended on-site treatment sessions and were provided with strategies for behavior modification, physical activity increase, and portion control.^25^ The intervention resulted in significantly greater weight loss and improvement in glycemic control, lipids, and blood pressure compared to the control group, Diabetes Support and Education (DSE),^26,27^ but did not reduce cardiovascular morbidity and mortality.^28^ Individual-level variables such as age, race, sex, and education were collected at baseline as part of the main Look AHEAD study. Additionally, income, weight, body mass index (BMI), HbA1c, blood pressure, and lipids were gathered at baseline and every year over 4 years.

For this ancillary study, participants from four Look AHEAD study sites (Baltimore, New York, Pittsburgh, and Philadelphia) had their baseline home addresses geocoded and linked to their respective 2000 US census tracts. Neighborhood deprivation scores were estimated using the procedure described by Diez-Roux et al.^29,30^. A summary neighborhood deprivation score was constructed using the 2000 U.S. Census data. The score was obtained by summing Z score for six variables representing indicators of wealth/income, education, and occupation.^29^ Finally, neighborhood scores were categorized into tertiles.^30^ The 2000 US Census data was also used to obtain other neighborhood variables, including the proportion of Black residents and percentage of residents living below the poverty level.

The primary outcomes of interest were the interactions between the intervention assignment and the following variables: weight, HbA1c, systolic blood pressure (SBP), diastolic blood pressure (DBP), total cholesterol, LDL cholesterol, HDL cholesterol, and triglycerides over 4 years. We conducted an exploratory analysis which showed a normal distribution of the outcomes except for triglycerides, which was then log transformed for our analyses. The percentage of missing data was less than 10% and randomly distributed, so no imputation was employed. We used a mixed linear model with random intercept to assess the difference in difference in intervention effects across neighborhood deprivation score tertiles using an interaction term between neighborhood score tertile, study year, and randomization arm. The model was adjusted for site. We compared models with the neighborhood deprivation scores as continuous variables, categorical variables (tertiles), and combining tertiles, and finally selected the model with the neighborhood deprivation tertile which had the lower AIC. The mean outcomes and the coefficient for the interaction term were estimated for each tertile. Finally, a global test was used to estimate the significance of the interaction term for each year. Since we conducted multiple analyses using the same dataset, encompassing eight outcomes (weight, HbA1c, SBP, DBP, LDL, HDL, total cholesterol, and triglycerides), we applied a Bonferroni-adjusted alpha level of 0.006.

As sensitivity analyses, we examined differences in the outcomes across tertiles of the percentage of residents below poverty level and the proportion of Black residents, instead of the neighborhood deprivation index. The proportion of Black residents has been previously used in the literature as a proxy for neighborhood deprivation and socio-economic disparities.^18^ All analyses were conducted using Stata version 17.

### Data and Resource Availability

The data from the main Look AHEAD study are available publicly through the NIDDK data repository. The data generated for the ancillary study are not publicly available as it was not required to be shared and contains potentially identifiable information; these are available from the ancillary study PI (TGW) upon request. The study met Johns Hopkins’ guidelines for protection of human subjects concerning their safety and privacy.

## RESULTS

### Baseline Characteristics

Of 1298 possible participants, 93.45% were successfully geocoded. Among these, 602 participants were randomized to the ILI group and 611 the DSE group (Table 1). The mean age was 60 years, with 41% male and 65% self-identifying as White, 26% Black, and 4% Hispanic. A majority, 84%, had a college degree or greater, and 75% reported an annual income over $40,000. The mean A1c was 7.3% and mean BMI was 36.1 kg/m^2^ at baseline. Apart from the mean diastolic blood pressure (69.9 in ILI vs 71.2 in DSE), there were no statistically significant differences between baseline variables in the ILI and DSE groups in this study subsample. This subsample was slightly older than the main Look AHEAD study sample (mean age 60 vs 59 years) and had a higher proportion of participants who identified as Black (26% vs 15.6% in the main study sample).^31^

**Table 1:** Baseline Characteristics of Participants in the ILI and DSE Groups

Characteristic	ILI	DSE	*P* value
	*N* = 602	*N*=611	
Site			
Baltimore	145 (24.1)	155 (25.4)	0.97
New York	150 (24.9)	150 (24.5)	
Philadelphia	147 (24.4)	146 (23.9)	
Pittsburg	160 (26.6)	160 (26.2)	
Female	357 (59.3)	355 (58.1)	0.67
Age (yrs.)	60.4 (6.8)	60.2 (6.6)	0.62
Race			
Non-Hispanic White	395 (65.6)	396 (64.8)	0.74
Black	156 (25.9)	168 (27.5)	
Hispanic	25 (4.2)	27 (4.4)	
Other	26 (4.3)	20 (3.3)	
Education level			
Less than high school	13 (2.2)	9 (1.5)	0.28
High school or equivalent	94 (15.6)	79 (12.9)	
Vocational/some college/associate degree	207 (34.4)	198 (32.4)	
Bachelor's degree	85 (14.1)	86 (14.1)	
Post-graduate degree	203 (33.7)	239 (39.1)	
Income			
Under $20,000	45 (8.8)	34 (6.5)	0.56
$20,000–$39,999	91 (17.7)	95 (18.0)	
$40,000–$59,999	111 (21.6)	104 (19.7)	
$60,000–$79,999	88 (17.1)	96 (18.2)	
>$80,000	179 (34.8)	198 (37.6)	
Deprivation Index	0.1 (5.1)	−0.2 (4.9)	0.37
Weight (kg)	101.1 (18.1)	102.7 (17.9)	0.13
BMI (kg/m^2^)	36.0 (6.0)	36.2 (5.5)	0.65
SBP (mm/Hg)	128.8 (16.9)	129.6 (17.0)	0.44
DBP (mm/Hg)	69.9 (10.0)	71.2 (9.6)	0.019
HbA1c (%)	7.2 (1.1)	7.4 (1.3)	0.11
Cholesterol (mg/dL)	191.6 (37.6)	190.5 (36.1)	0.6
HDL cholesterol (mg/dL)	45.1 (12.4)	45.1 (12.3)	1
LDL cholesterol (mg/dL)	115.2 (32.4)	113.0 (30.5)	0.22
Triglyceride (mg/dL)	158.0 (94.9)	164.2 (110.7)	0.3

Values shown are means ± SDs or frequency counts (with percentages)

The neighborhood deprivation score ranged between −12.03 and 18.69. The mean deprivation score was −5.34 in tertile 1 (Mi: −12.04; Max −2.61), −0.4 in tertile 2 (Min −2.59 to Max 2) and 5.7 in tertile 3 (Min 2.01; Max 18.69). The lowest tertile (tertile 1) represented the most deprived neighborhoods and the highest tertile (tertile 3) represented the least deprived areas. Participants residing in the most-deprived neighborhoods were disproportionately women and Black, and tended to have lower education attainment and lower household income when compared to those from the least disadvantaged neighborhoods (Table 2). However, the average weight, blood pressure, HbA1c, total cholesterol, HDL cholesterol, LDL cholesterol, and triglycerides at baseline were similar across the tertiles of neighborhood deprivation score.

**Table 2:** Baseline Characteristics by Tertile of Neighborhood Deprivation Score

Tertile of neighborhood deprivation score	T1 (lowest)		T2 (middle)		T3 (highest)	
	Min= −12.03	Max= −2.61	Min= −2.59	Max= 2.00	Min= 2.01	Max= 18.69
Characteristics	**ILI**	**DSE**	**ILI**	**DSE**	**ILI**	**DSE**
	*N*=200	*N*=205	*N*=195	*N*=210	*N*=207	*N*=196
Female (%)	71.0%	76.1%	56.9%	51.9%	50.2%	45.9%
Mean age (yrs.)	59.5 (6.9)	60.2 (6.6)	60.6 (6.5)	60.0 (6.9)	61.0 (6.8)	60.3 (6.4)
Race %						
White	39.5%	36.1%	77.4%	77.6%	79.7%	81.1%
Black	49.5%	55.6%	15.4%	16.2%	13.0%	10.2%
Hispanic	6.0%	5.9%	3.6%	2.9%	2.9%	4.6%
Other	5.0%	2.4%	3.6%	3.3%	4.3%	4.1%
Family income (%)						
< $20,000	17.7%	10.7%	4.4%	7.0%	3.9%	1.7%
$20,000–$39,999	26.3%	29.9%	17.5%	18.7%	9.5%	5.6%
$40,000–$79,999	40.6%	45.2%	40.0%	35.1%	35.8%	33.5%
≥ $80,000	15.4%	14.1%	38.1%	39.2%	50.8%	59.2%
Education level (%)						
≤ High school or less	26.0%	22.0%	19.0%	12.9%	8.7%	8.2%
≥ Some college	54.5%	54.6%	47.2%	51.9%	44.0%	32.1%
Graduate degree	19.5%	23.4%	33.8%	35.2%	47.3%	59.7%
Mean weight (kg)	101.1 (18.7)	101.3 (16.9)	100.4 (17.7)	104.1 (18.8)	101.8 (17.9)	102.6 (18.0)
Mean BMI	37.2 (6.7)	36.9 (5.5)	35.5 (5.4)	36.3 (5.8)	35.4 (5.7)	35.2 (4.9)
Mean HbA1c (%)	7.3 (1.1)	7.4 (1.3)	7.3 (1.1)	7.4 (1.2)	7.1 (1.0)	7.2 (1.2)

*T1* lowest tertile, indicating greatest deprivation; *ILI* intensive lifestyle intervention; *DSE* diabetes support and education

### Interaction of Treatment with Neighborhood Deprivation

#### Weight Loss

**Figure 1:**
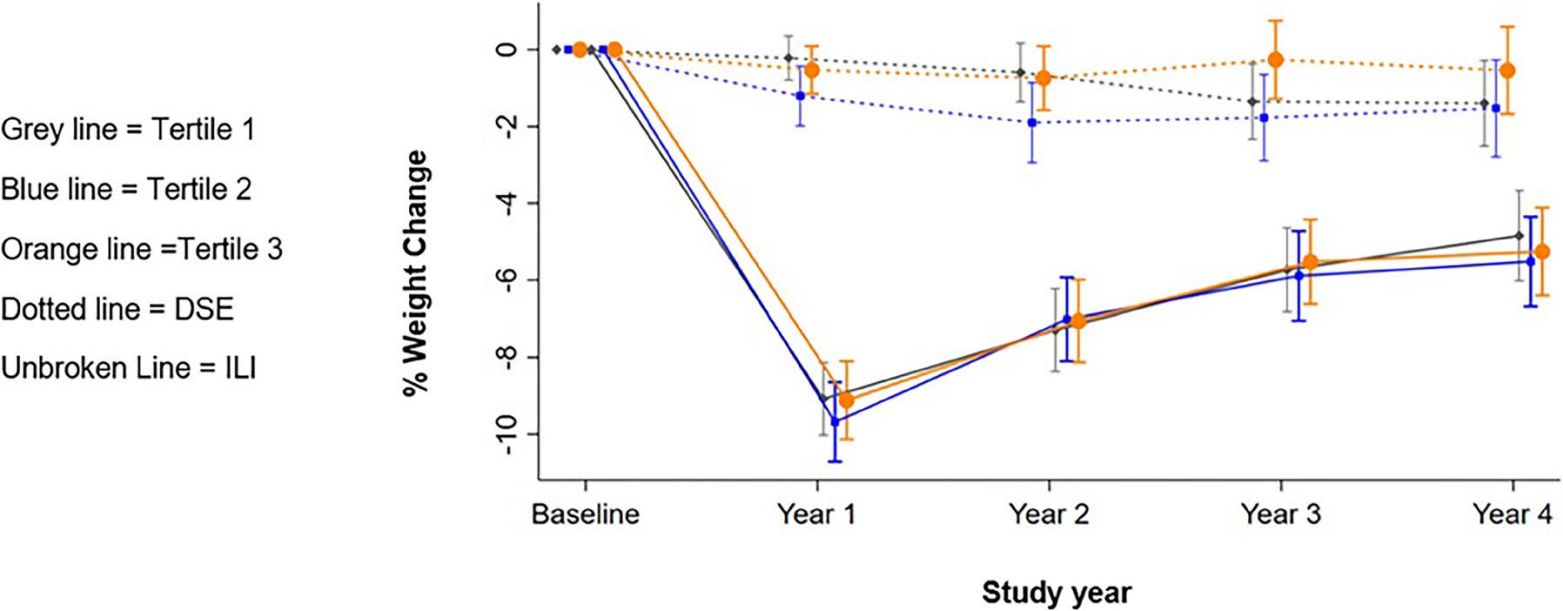
Change in weight over 4 years by neighborhood deprivation score tertile in ILI and DSE groups.

Table 3 shows the mean change in weight and HbA1c across each tertile of neighborhood deprivation score at year 1 and at year 4. At year 1, on average ILI participants in tertile 1 lost 9.33% (95%CI −10.21 to −8.43) of their initial weight and those in tertile 3 lost 9.46% of their baseline weight (95%CI −10.34 to −8.58). In the DSE group, the mean weight loss was −0.26 (95%CI −1.24 to 0.73) in tertile 1 and −0.57 (95%CI −1.55 to 0.43) in tertile 3. The changes in weight by arm and neighborhood deprivation score over the 4 years are shown in Fig. 1. The difference in difference between tertile and intervention arm was not statistically significant (*p*=0.94).

**Table 3:** Change in Weight and HbA1c by Tertile of Neighborhood Deprivation Score and Year

	ILI	DSE	*p* value*
% weight change			
Year 1			0.94
T1	−9.33(−10.21 to −8.43)	−0.26(−1.24 to 0.73)	
T2	−10.02(−10.91to −9.12)	−1.38(−2.34 to −0.41)	
T3	−9.46(−10.34 to −8.58)	−0.57(−1.55 to 0.43)	
Year 4			0.25
T1	−5.12(−6.07 to −4.17)	−1.74(−2.71 to −0.75)	
T2	−5.76(−6.71 to −4.79)	−2.00(−2.96 to −1.03)	
T3	−5.75(−6.68 to −4.81)	−0.77(−1.78 to 0.26)	
HbA1c (%)			
Year 1			0.75
T1	−0.71(−0.87 to −0.56)	−0.14(−0.29 to 0.02)	
T2	−0.69(−0.85 to −0.53)	−0.20(−0.36 to −0.05)	
T3	−0.58(−0.74 to −0.43)	−0.12(−0.28 to 0.03)	
Year 4			0.37
T1	−0.27(−0.43 to −0.12)	−0.11(−0.27 to 0.05)	
T2	−0.30(−0.46 to −0.14)	−0.31(−0.47 to −0.15)	
T3	−0.27(−0.43 to −0.11)	−0.32(−0.48 to −0.16)	

*ILI* intensive lifestyle intervention; *DSE* diabetes support and education; *T1* lowest tertile, indicating greatest deprivation; Values shown are means (95% confidence intervals); Pooled treatment effect for each year statistically significant at the p = 0.006 level

^*^p value for the interaction term between tertile x randomization x study year

At year 4, on average, ILI participants in tertile 1 lost −5.12% (95%CI −6.06 to −4.17) of their baseline weight and those in tertile 3 lost −5.75% (95%CI −6.68 to −4.81). In the DSE group, the mean weight loss was 1.74% (95%CI −2.71 to 0.75) in tertile 1 and −0.76 (95%CI −1.78 to 0.26) in tertile 3. The difference in difference between tertile and intervention arm was not statistically significant (*p*=0.25).

#### Glycemic Control

At year 1, the HbA1c for ILI participants in tertile 1 decreased by 0.71 (95%CI −0.87 to −0.56) in comparison to baseline; in tertile 3, it decreased by 0.58 (95%CI −0.74 to −0.43). In the DSE group in tertile 1, the HbA1c decreased by 0.14 (95%CI −0.29 to 0.02); in tertile 3, it decreased by 0.12 (95%CI −0.28 to 0.03). The difference in difference between tertile and intervention arm was not statistically significant, *p*=0.75. At year 4, the HbA1c also decreased similarly for both ILI and DSE participants across tertiles. The difference in difference between tertile and intervention arm was not statistically significant (*p*=0.37).

#### Other Outcomes and Deprivation Measures

Blood pressure and cholesterol levels also improved (Supplemental Materials Table S1) without any significant interactions across deprivation levels. Overall, we found no significant difference in effect on any of our specified outcomes across the neighborhood deprivation score tertiles between ILI and DSE for each year (*p*≥0.01). Analysis across neighborhood racial composition and poverty level showed similar results (Supplemental Materials Tables S2, S3).

## DISCUSSION

In summary, we found that neighborhood socioeconomic deprivation did not moderate the effect of an intensive lifestyle intervention on several clinical outcomes in participants with overweight/obesity and type 2 diabetes. Regardless of the level of deprivation, individuals in the ILI group lost substantial weight at year 1 and regained some weight over the course of 4 years. Additionally, other clinical and biological markers such as HbA1c, blood pressure, and lipids all improved in the intervention arm, with no discernible impact of neighborhood deprivation on the ILI’s effectiveness. Our findings were the same when alternative neighborhood measures, such as neighborhood racial composition and poverty level, were considered. These results align with those of the primary Look AHEAD study.

Our study contributes to the scientific literature regarding the role of the patient’s environment in health. While the correlation between poor living conditions and poor diabetes control is well-established,^7^ our findings suggest that, for individuals with obesity and diabetes, neighborhood socioeconomic deprivation does not influence the effectiveness of an intensive lifestyle intervention. It is important to reiterate that our focus was on the concept of neighborhood socioeconomic deprivation, as defined by indicators of wealth, education, and occupation.^29^

To our knowledge, this is the first study to specifically investigate the influence of neighborhood deprivation on the effectiveness of an intensive lifestyle intervention in patients with both overweight and diabetes. Prior studies yielded mixed results. For instance, Zenk et al. reported similar patterns of weight loss and regain in their quasi-experimental study using a generalized difference-in-differences design with an inverse propensity score matched comparison group to evaluate a weight management program for US military veterans.^32^ They found that the built environment did not influence the effectiveness.^32^ In contrast, Jiang et al. found that participants from neighborhoods with lower socioeconomic scores had less favorable outcomes in a diabetes prevention intervention for American Indians and Alaska Natives. However, their study was limited by a lack of racial/ethnic diversity.^33^ Mendez et al. highlighted the moderating effect of neighborhood racial composition in a weight loss intervention but found no evidence of the influence of neighborhood socioeconomic status.^18^ They did find that that study participants living in racially diverse neighborhoods (in which 25–75% of the residents identified as Black) were more likely to lose weight.^18^ This study had a relatively small size (127 participants) and a large proportion of White participants (81% of the study population).^18^ Saint Onge et al. also examined the role of socioeconomic neighborhood disadvantage and other factors of obesogenic environment in a weight loss trial. They found no influence; however, their study provided new insights about the importance of the modality of intervention delivery.^34^ In that study, participants were randomly assigned to a group receiving the intervention either in-clinic one-on-one sessions, in clinic with group sessions, or by telephone call. Participants assigned to the group sessions lost less weight when they lived in a neighborhood with dollar stores.^34^ Overall, these studies vary in their measures of neighborhood environment, analysis methods, and populations studied, making direct comparisons difficult.

A possible explanation for our results includes the efficacy of the Look AHEAD trial in addressing neighborhood socioeconomic challenges. Even though residents of poor neighborhoods are more likely to experience limited access to nutritious food,^35^ higher exposure to unhealthy dietary options,^36^ and suboptimal built environments, the Look AHEAD ILI may have effectively mitigated these obstacles, by virtue of several key components. First, participants received crucial information and tools about diabetes management, nutrition, and exercise, which may have empowered them to make informed decisions regarding their lifestyles, thus helping them navigate the complexities of their environments. In addition, the presence of group support may have allowed participants to share experiences and strategies, fostering a sense of community and motivation that transcended the barriers presented by their neighborhoods.

An additional factor that could account for our findings relates to the characteristics of the participants in the Look AHEAD trial. The cohort was characterized by very high levels of motivation and commitment. Participants voluntarily consented to engage in a rigorous long-term study with an intensive intervention an over a decade of longitudinal follow-up, and study adherence rates were high.^23^ Additionally, as part of the trial’s entry requirement, participants completed a thorough behavioral screening,^37^ which included a 2-week run-in period, during which they recorded daily their dietary habits and physical activity.^38^ These protocols may have naturally selected for individuals who were more willing and able to invest substantial time and effort in managing their health than the general population. Self-efficacy is predictive of higher adherence not only to the study protocol (increase of physical activity and diet improvement) but also to medication adherence and blood glucose level monitoring.^39^ As a result, the study participants may have been more able than many to overcome their respective neighborhood barriers.

Another crucial aspect to consider in our study pertains to the use of the neighborhood deprivation score as a metric to capture disparities in neighborhood socioeconomic status. This score has been widely employed in numerous studies to effectively gauge socioeconomic disparities^29,33^ and serves as a valuable tool for classifying and comparing neighborhoods. However, its utility hinges on the assumption that deprivation is present uniformly within these neighborhoods. When this assumption is met, the neighborhood deprivation score offers valuable insights, yet it can be misleading when applied in contexts where deprivation may not be uniform. In our sample, we observed that the lower deprivation tertile had a higher percentage of female and Black participants, as well as lower educational attainment and lower household income, aligning with what one would typically expect when assessing disparities using the neighborhood deprivation score in the US context. Nonetheless, the study cohort as a whole displayed a notably high level of education and income: 84% had a college degree or greater, and 75% reported an annual income over $40,000. Even in the most deprived neighborhoods within the intervention arm, a substantial proportion of participants held college degrees or higher (at least 74%), and a noteworthy 15.4% reported an income of at least $80,000. Conversely, those with a high school degree or less comprised only 26%, and only 17.7% reported a family income below $20,000. These observations raise the question of whether the term “deprivation” in this context holds the same significance as it does in other, less affluent populations. While our study did indeed capture disparities relative to the specific group examined, it likely does not truly reflect the deprivation levels that exist across the US.

The study has limitations that must be considered. First, it relies on the 2000 US Census data to characterize the neighborhoods, potentially not capturing changes in the neighborhood’s demographic and economic status after that time. Moreover, the deprivation scores were derived from participants’ baseline addresses, without accounting for potential residential changes during the 4-year analysis period. Finally, the study inclusion criteria led to the selection of a predominantly highly educated and high-income population.

Strengths of this study include that it draws upon the well-established and rigorously designed Look AHEAD trial, a randomized controlled trial, lending strong credibility to its findings. Additionally, the study benefits from a large and diverse sample of participants, totaling 1213 individuals, with varying demographic characteristics. This diversity enhances the generalizability of the results to broader populations. Moreover, the study’s comprehensive assessment of clinical outcomes, including weight, HbA1c, blood pressure, and lipid profiles, ensures a thorough evaluation of the intervention’s effects on participants’ health.

In conclusion, this study highlights the complexity of the relationship between neighborhood deprivation and the effect of lifestyle intervention on health outcomes. The results suggest that the Look AHEAD trial’s intensive lifestyle intervention was successful in mitigating the challenges posed by neighborhood socioeconomic factors. The provision of essential information, education, and group support and also a high level of commitment and motivation likely played pivotal roles in helping participants overcome barriers associated with their neighborhood environments. However, it is essential to recognize that these findings may not necessarily apply to individuals with the lowest income and educational attainment, who remain underrepresented in clinical trials.

## Supplementary Information

Below is the link to the electronic supplementary material.

Supplementary file1 (DOCX 45.6 KB)
