# Supplementary material for: Influence of Neighborhood Socioeconomic Deprivation on Effectiveness of an Intensive Lifestyle Intervention
Source: J Gen Intern Med. 2025 Jan 2;40(8):1728–34. doi: 10.1007/s11606-024-09232-5 (PMC12119450; doi:10.1007/s11606-024-09232-5)
Supplement: Supplementary file 1 — Supplementary file1 (DOCX 45.6 KB) [file 11606_2024_9232_MOESM1_ESM.docx]

**Supplemental Material**

**Table S1.** Change in outcome variables by Tertile of Neighborhood Deprivation Score and Year using the socioeconomic deprivation score.

|  | **ILI** | **DSE** | **p value*** |
| --- | --- | --- | --- |
| **SBP (mmHg)** |  |  |  |
| **Year 1** |  |  | 0.87 |
| **T1** | -6.28(-8.67 to -3.89) | -1.28(-3.67 to 1.12) |  |
| **T2** | -6.42(-8.84 to -4.01) | -2.24(-4.61 to 0.13) |  |
| **T3** | -5.57(-7.93 to -3.21) | -1.85(-4.27 to 0.58) |  |
| **Year 4** |  |  | 0.28 |
| **T1** | -0.52(-2.95 to 1.91) | -1.75(-4.17 to 0.67) |  |
| **T2** | -4.29(-6.76 to -1.82) | -1.62(-4.00 to 0.77) |  |
| **T3** | -2.09(-4.51 to 0.32) | -1.72(-4.21 to 0.77) |  |
| **DBP (mmHg)** |  |  |  |
| **Year 1** |  |  | 0.36 |
| **T1** | -1.82(-3.00 to -0.64) | -1.04(-2.22 to 0.15) |  |
| **T2** | -3.01(-4.20 to -1.81) | -0.97(-2.14 to 0.21) |  |
| **T3** | -1.83(-3.00 to -0.66) | -1.45(-2.65 to -0.25) |  |
| **Year 4** |  |  | 0.17 |
| **T1** | -0.55(-1.75 to 0.65) | -1.05(-2.25 to 0.15) |  |
| **T2** | -2.71(-3.93 to -1.48) | -2.29(-3.47 to -1.10) |  |
| **T3** | -1.13(-2.32 to 0.07) | -3.02(-4.25 to -1.79) |  |
|  |  |  |  |
|  | **ILI** | **DSE** | **p value*** |
| **Cholesterol (mg/dl)** |  |  |  |
| **Year 1** |  |  | 0.63 |
| **T1** | -5.76(-10.70 to -0.83) | -6.91(-11.88 to -1.94) |  |
| **T2** | -7.10(-12.09 to -2.12) | -8.93(-13.82 to -4.03) |  |
| **T3** | -12.66(-17.52 to -7.81) | -9.97(-14.98 to -4.97) |  |
| **Year 4** |  |  | 0.96 |
| **T1** | -12.44(-17.48 to -7.41) | -15.63(-20.65 to-10.60) |  |
| **T2** | -21.64(-26.73 to -16.56) | -26.16(-31.12 to-21.21) |  |
| **T3** | -23.85(-28.82 to -18.88) | -27.11(-32.25 to-21.96) |  |
| **Log10 Trigl** |  |  |  |
| **Year 1** |  |  | 0.76 |
| **T1** | -0.06(-0.08 to -0.03) | -0.02(-0.04 to 0.01) |  |
| **T2** | -0.10(-0.12 to -0.07) | -0.04(-0.06 to -0.01) |  |
| **T3** | -0.07(-0.10 to -0.05) | -0.02(-0.05 to 0.00) |  |
| **Year 4** |  |  | 0.18 |
| **T1** | -0.03(-0.06 to -0.00) | -0.06(-0.09 to -0.04) |  |
| **T2** | -0.07(-0.10 to -0.05) | -0.06(-0.09 to -0.04) |  |
| **T3** | -0.07(-0.09 to -0.04) | -0.06(-0.08 to -0.03) |  |
|  |  |  |  |
|  | **ILI** | **DSE** | **p value*** |
| **LDL Cholesterol**  **(mg/dl)** |  |  |  |
| **Year 1** |  |  | 0.86 |
| **T1** | -5.84(-10.04 to -1.63) | -4.90(-9.13 to -0.66) |  |
| **T2** | -4.38(-8.63 to -0.13) | -5.69(-9.87 to -1.52) |  |
| **T3** | -9.86(-13.99 to -5.72) | -10.58(-14.85 to-6.32) |  |
| **Year 4** |  |  | 0.61 |
| **T1** | -14.60(-18.89 to -10.31) | -13.33(-17.61to-9.04) |  |
| **T2** | -19.41(-23.75 to -15.07) | -22.04(-26.27 to-17.82) |  |
| **T3** | -22.95(-27.19 to -18.71) | -25.26(-29.64 to-20.87) |  |
| **HDL Cholesterol**  **(mg/dl)** |  |  |  |
| **Year 1** |  |  | 0.04 |
| **T1** | 3.89(2.82 to 4.96) | 0.89(-0.19 to 1.97) |  |
| **T2** | 4.02(2.94 to 5.11) | 0.95(-0.12 to 2.01) |  |
| **T3** | 2.58(1.53 to 3.64) | 1.97(0.88 to 3.05) |  |
| **Year 4** |  |  | 0.83 |
| **T1** | 4.97(3.88 to 6.06) | 2.94(1.84 to 4.03) |  |
| **T2** | 3.25(2.14 to 4.35) | 1.78(0.71 to 2.86) |  |
| **T3** | 4.00(2.92 to 5.08) | 2.58(1.46 to 3.69) |  |

ILI: Intensive Lifestyle Intervention; DSE: Diabetes Support and Education; T1: Lowest tertile, indicating greatest deprivation; Values shown are means (95% confidence intervals); Pooled treatment effect for each year statistically significant at the p < 0.006 level

*p value for the interaction term between the **Tertile× Randomization Arm× Study Year**

**Table S2.** Change in outcome variables by Tertile of the percentage of residents below the poverty level and Year.

|  | **ILI** | **DSE** | **p value*** |
| --- | --- | --- | --- |
| **% Weight Change** | | | |
| **Year 1** |  |  | 0.55 |
| **T1** | -9.91(-10.80 to -9.01) | -0.73(-1.69 to 0.25) |  |
| **T2** | -9.79(-10.65 to -8.91) | -1.59(-2.58 to -0.60) |  |
| **T3** | -9.09(-9.99 to -8.19) | 0.04(-0.93 to 1.03) |  |
| **Year 4** |  |  | 0.17 |
| **T1** | -5.76(-6.70 to -4.80) | -0.89(-1.86 to 0.10) |  |
| **T2** | -5.17(-6.09 to -4.23) | -2.15(-3.15 to -1.14) |  |
| **T3** | -5.75(-6.70 to -4.78) | -1.56(-2.53 to -0.57) |  |
| **HbA1c** |  |  |  |
| **Year 1** |  |  | 0.15 |
| **T1** | -0.71(-0.87 to -0.56) | -0.05(-0.20 to 0.11) |  |
| **T2** | -0.58(-0.73 to -0.43) | -0.22(-0.38 to -0.06) |  |
| **T3** | -0.69(-0.85 to -0.54) | -0.20(-0.36 to -0.05) |  |
| **Year 4** |  |  | 0.03 |
| **T1** | -0.35(-0.51 to -0.19) | -0.25(-0.40 to -0.09) |  |
| **T2** | -0.24(-0.39 to -0.08) | -0.45(-0.61 to -0.29) |  |
| **T3** | -0.26(-0.42 to -0.09) | -0.05(-0.21 to 0.10) |  |
|  |  |  |  |
|  | **ILI** | **DSE** | **p value*** |
| **SBP(mmHg)** |  |  |  |
| **Year 1** |  |  | 0.49 |
| **T1** | -6.93(-9.33 to -4.52) | -1.44(-3.81 to 0.93) |  |
| **T2** | -6.32(-8.66 to -3.97) | -1.63(-4.07 to 0.82) |  |
| **T3** | -5.00(-7.41 to -2.59) | -2.32(-4.69 to 0.05) |  |
| **Year 4** |  |  | 0.79 |
| **T1** | -2.82(-5.27 to -0.36) | -1.49(-3.89 to 0.90) |  |
| **T2** | -3.28(-5.66 to -0.90) | -2.61(-5.09 to -0.13) |  |
| **T3** | -0.67(-3.15 to 1.80) | -1.02(-3.43 to 1.38) |  |
| **DBP** |  |  |  |
| **Year 1** |  |  | 0.35 |
| **T1** | -2.69(-3.88 to -1.50) | -0.78(-1.95 to 0.40) |  |
| **T2** | -2.34(-3.50 to -1.19) | -1.26(-2.47 to -0.05) |  |
| **T3** | -1.59(-2.78 to -0.40) | -1.42(-2.59 to -0.24) |  |
| **Year 4** |  |  | 0.54 |
| **T1** | -1.94(-3.15 to -0.72) | -2.42(-3.60 to -1.23) |  |
| **T2** | -1.76(-2.93 to -0.58) | -3.20(-4.43 to -1.97) |  |
| **T3** | -0.63(-1.85 to 0.60) | -0.75(-1.94 to 0.44) |  |
|  |  |  |  |
|  | **ILI** | **DSE** | **p value*** |
| **Cholesterol**  **(mg/dl)** |  |  |  |
| **Year 1** |  |  | 0.78 |
| **T1** | -8.91(-13.85 to -3.98) | -7.73(-12.61 to -2.85) |  |
| **T2** | -10.21(-15.05 to -5.37) | -12.37(-17.43 to-7.30) |  |
| **T3** | -6.51(-11.49 to -1.53) | -5.96(-10.86 to -1.05) |  |
| **Year 4** |  |  | 0.15 |
| **T1** | -20.69(-25.74 to -15.63) | -27.83(-32.76 to-22.89) |  |
| **T2** | -23.35(-28.25 to -18.45) | -29.67(-34.86 to-24.49) |  |
| **T3** | -13.59(-18.71 to -8.46) | -11.68(-16.67 to-6.69) |  |
| **Log10 Trigl** |  |  |  |
| **Year 1** |  |  | 0.18 |
| **T1** | -0.09(-0.11 to -0.06) | -0.01(-0.04 to 0.01) |  |
| **T2** | -0.09(-0.11 to -0.06) | -0.05(-0.07 to -0.02) |  |
| **T3** | -0.06(-0.08 to -0.03) | -0.03(-0.05 to -0.00) |  |
| **Year 4** |  |  | 0.06 |
| **T1** | -0.08(-0.10 to -0.05) | -0.05(-0.08 to -0.03) |  |
| **T2** | -0.06(-0.08 to -0.03) | -0.09(-0.12 to -0.06) |  |
| **T3** | -0.03(-0.06 to -0.00) | -0.05(-0.07 to -0.02) |  |
|  |  |  |  |
|  | **ILI** | **DSE** | **p value*** |
| **LDL Cholesterol**  **(mg/dl)** |  |  |  |
| **Year 1** |  |  | 0.45 |
| **T1** | -6.54(-10.75 to -2.33) | -8.14(-12.30 to -3.98) |  |
| **T2** | -7.31(-11.43 to -3.19) | -9.51(-13.83 to -5.19) |  |
| **T3** | -6.38(-10.62 to -2.13) | -3.60(-7.78 to 0.59) |  |
| **Year 4** |  |  | 0.04 |
| **T1** | -19.03(-23.34 to -14.72) | -25.35(-29.56 to-21.15) |  |
| **T2** | -22.91(-27.09 to -18.73) | -25.36(-29.78 to-20.94) |  |
| **T3** | -14.73(-19.09 to -10.36) | -10.03(-14.29 to-5.78) |  |
| **HDL Cholesterol**  **(mg/dl)** |  |  |  |
| **Year 1** |  |  | 0.49 |
| **T1** | 3.54(2.47 to 4.62) | 1.19(0.12 to 2.25) |  |
| **T2** | 3.03(1.98 to 4.08) | 1.52(0.41 to 2.62) |  |
| **T3** | 3.89(2.81 to 4.98) | 1.10(0.02 to 2.17) |  |
| **Year 4** |  |  | 0.54 |
| **T1** | 4.46(3.35 to 5.56) | 2.12(1.04 to 3.19) |  |
| **T2** | 3.71(2.64 to 4.77) | 2.53(1.40 to 3.66) |  |
| **T3** | 4.08(2.96 to 5.20) | 2.67(1.58 to 3.76) |  |

ILI: Intensive Lifestyle Intervention; DSE: Diabetes Support and Education; T1: Lowest tertile, indicating greatest deprivation; Values shown are means (95% confidence intervals); Pooled treatment effect for each year statistically significant at the p < 0.006 level

*p value for the interaction term between the **Tertile× Randomization Arm× Study Year**

**Table S3.** Change in outcome variables by Tertile of percentage of Black residents and Year.

|  | **ILI** | **DSE** | **p value*** |
| --- | --- | --- | --- |
| **% Weight Change** |  |  |  |
| **Year 1** |  |  | **0.23** |
| **T1** | -9.96(-10.83 to -9.09) | -0.87(-1.88 to 0.14) |  |
| **T2** | -10.50(-11.36 to -9.62) | -1.11(-2.08 to -0.14) |  |
| **T3** | -8.24(-9.16 to -7.31) | -0.26(-1.22 to 0.71) |  |
| **Year 4** |  |  | 0.39 |
| **T1** | -5.69(-6.61 to -4.76) | -1.46(-2.47 to -0.44) |  |
| **T2** | -6.09(-7.03 to -5.15) | -1.56(-2.54 to -0.57) |  |
| **T3** | -4.79(-5.76 to -3.81) | -1.52(-2.48 to -0.56) |  |
| **HbA1c** |  |  |  |
| **Year 1** |  |  | 0.05 |
| **T1** | -0.74(-0.89 to -0.59) | -0.12(-0.28 to 0.04) |  |
| **T2** | -0.70(-0.86 to -0.55) | -0.10(-0.26 to 0.06) |  |
| **T3** | -0.52(-0.68 to -0.37) | -0.24(-0.40 to -0.09) |  |
| **Year 4** |  |  | 0.19 |
| **T1** | -0.37(-0.52 to -0.21) | -0.17(-0.33 to -0.01) |  |
| **T2** | -0.33(-0.49 to -0.17) | -0.37(-0.53 to -0.22) |  |
| **T3** | -0.12(-0.29 to 0.04) | -0.19(-0.35 to -0.03) |  |
|  |  |  |  |
|  | **ILI** | **DSE** | **p value*** |
| **SBP(mmHg)** |  |  |  |
| **Year 1** |  |  | 0.47 |
| **T1** | -6.82(-9.17 to -4.47) | -1.79(-4.26 to 0.67) |  |
| **T2** | -6.30(-8.67 to -3.93) | -1.08(-3.46 to 1.31) |  |
| **T3** | -5.05(-7.50 to -2.59) | -2.52(-4.86 to -0.17) |  |
| **Year 4** |  |  | 0.86 |
| **T1** | -2.96(-5.34 to -0.57) | -1.91(-4.41 to 0.59) |  |
| **T2** | -2.60(-5.04 to -0.17) | -1.79(-4.22 to 0.63) |  |
| **T3** | -1.18(-3.68 to 1.32) | -1.40(-3.78 to 0.97) |  |
| **DBP** |  |  |  |
| **Year 1** |  |  | 0.13 |
| **T1** | -2.75(-3.91 to -1.59) | -1.08(-2.31 to 0.14) |  |
| **T2** | -2.33(-3.51 to -1.16) | -0.50(-1.68 to 0.68) |  |
| **T3** | -1.48(-2.69 to -0.27) | -1.84(-3.01 to -0.68) |  |
| **Year 4** |  |  | 0.34 |
| **T1** | -2.04(-3.22 to -0.86) | -1.91(-3.15 to -0.67) |  |
| **T2** | -0.99(-2.19 to 0.22) | -2.64(-3.84 to -1.44) |  |
| **T3** | -1.27(-2.51 to -0.04) | -1.76(-2.94 to -0.58) |  |
|  |  |  |  |
|  | **ILI** | **DSE** | **p value*** |
| **Cholesterol**  **(mg/dl)** |  |  |  |
| **Year 1** |  |  | 0.90 |
| **T1** | -10.33(-15.16to -5.49) | -10.58(-15.67 to -5.48) |  |
| **T2** | -7.99(-12.89 to -3.09) | -9.22(-14.16 to -4.27) |  |
| **T3** | -7.28(-12.35 to -2.21) | -6.23(-11.09 to -1.37) |  |
| **Year 4** |  |  | 0.30 |
| **T1** | -23.46(-28.38to -18.54) | -24.65(-29.83 to -19.47) |  |
| **T2** | -18.37(-23.41to -13.34) | -26.73(-31.76 to -21.71) |  |
| **T3** | -15.84(-21.01to -10.67) | -17.62(-22.58 to -12.67) |  |
| **Log10 Trigl** |  |  |  |
| **Year 1** |  |  | 0.09 |
| **T1** | -0.09(-0.12 to -0.07) | -0.01(-0.04 to 0.01) |  |
| **T2** | -0.08(-0.10 to -0.05) | -0.03(-0.06 to -0.01) |  |
| **T3** | -0.06(-0.08 to -0.03) | -0.03(-0.06 to -0.01) |  |
| **Year 4** |  |  | 0.01 |
| **T1** | -0.08(-0.11 to -0.06) | -0.04(-0.07 to -0.02) |  |
| **T2** | -0.05(-0.08 to -0.03) | -0.07(-0.10 to -0.05) |  |
| **T3** | -0.03(-0.06 to -0.00) | -0.07(-0.10 to -0.05) |  |
|  |  |  |  |
|  |  |  |  |
|  | **ILI** | **DSE** | **p value*** |
| **LDL Cholesterol**  **(mg/dl)** |  |  |  |
| **Year 1** |  |  | 0.28 |
| **T1** | -7.29(-11.42 to -3.17) | -10.50(-14.85 to -6.15) |  |
| **T2** | -5.85(-10.03 to -1.67) | -7.29(-11.51 to -3.08) |  |
| **T3** | -7.12(-11.44 to -2.80) | -3.66(-7.81 to 0.48) |  |
| **Year 4** |  |  | 0.27 |
| **T1** | -20.67(-24.86to -16.47) | -22.69(-27.11 to -18.28) |  |
| **T2** | -18.83(-23.12to -14.54) | -23.24(-27.52 to -18.96) |  |
| **T3** | -17.42(-21.82to -13.01) | -14.81(-19.03 to -10.59) |  |
| **HDL Cholesterol**  **(mg/dl)** |  |  |  |
| **Year 1** |  |  | 0.83 |
| **T1** | 3.25(2.20 to 4.30) | 0.73(-0.39 to 1.84) |  |
| **T2** | 3.87(2.81 to 4.93) | 1.56(0.49 to 2.63) |  |
| **T3** | 3.32(2.22 to 4.42) | 1.46(0.40 to 2.52) |  |
| **Year 4** |  |  | 0.47 |
| **T1** | 3.56(2.50 to 4.63) | 1.65(0.52 to 2.78) |  |
| **T2** | 4.75(3.66 to 5.84) | 2.55(1.46 to 3.64) |  |
| **T3** | 3.92(2.80 to 5.04) | 3.03(1.95 to 4.11) |  |
|  |  |  |  |

ILI: Intensive Lifestyle Intervention; DSE: Diabetes Support and Education; T1: Lowest tertile, indicating greatest deprivation; Values shown are means (95% confidence intervals); Pooled treatment effect for each year statistically significant at the p < 0.006 level

*p value for the interaction term between the **Tertile× Randomization Arm× Study Year**

**Authors:**

Mamadou Sy, Scott Pilla, Wendy Bennett, Hsin-Chieh Yeh, Kesha Baptiste-Roberts, Tiffany L. Gary-Webb, Dhananjay Vaidya, Jeanne M. Clark.

**Corresponding author:**

Dr. Mamadou Sy [msy@gmu.edu](mailto:jmclark@jhmi.edu)
